# Morphological and Physiological Stress Responses of Lettuce to Different Intensities of Continuous Light

**DOI:** 10.3389/fpls.2019.01440

**Published:** 2019-11-06

**Authors:** Lingyan Zha, Wenke Liu, Yubin Zhang, Chengbo Zhou, Mingjie Shao

**Affiliations:** ^1^Institute of Environment and Sustainable Development in Agriculture, Chinese Academy of Agricultural Sciences, Beijing, China; ^2^Key Lab of Energy Conservation and Waste Management of Agricultural Structures, Ministry of Agriculture and Rural Affairs, Beijing, China

**Keywords:** ascorbic acid, enzyme activity, reactive oxygen species, ascorbate–glutathione cycle, lipid peroxidation

## Abstract

In this study, specific dynamic changes in growth, oxidative stress, ascorbate metabolism, and chlorophyll fluorescence were monitored during 12 days in lettuce plants exposed to continuous light (CL) of different intensities: low light (LL, 100 μmol·m^−2^·s^−1^), medium light (ML, 200 μmol·m^−2^·s^−1^), and high light (HL, 300 μmol·m^−2^·s^−1^). Lettuce plants grown under CL of higher light intensity gained greater biomass, dry weight ratio, root/shoot ratio, and specific leaf FW, but not leaf area. Both the reactive oxygen species (ROS) production and the lipid peroxidation degree, measured in terms of the malondialdehyde (MDA) levels, were progressively enhanced by increasing the light intensity of CL. Overall, the pool sizes of ascorbate (AsA) and glutathione, as well as the activities of enzymes involved in AsA metabolism, had positive correlations with light intensity under CL. Ascorbate peroxidase and dehydroascorbate reductase presented the maximal and minimal responses to light intensity, respectively, among all the studied enzymes. After 6 days under CL, ML and HL intensity caused reversible photoinhibition, represented by lower values of maximum quantum efficiency (*F*_v _/*F*_m_), effective quantum yield (ΦPSII), and photochemical quenching (qP) and a higher value of non-photochemical quenching (qN). However, this photoinhibition recovered on day 12 with increasing of *F*_v _/*F*_m_, ΦPSII, and qP. Taken together, under ML and HL conditions, greater AsA level could help maintain photosynthetic efficiency by elevating excess excitation energy dissipation, though ROS accumulation and lipid peroxidation could not be prevented in the long-term. Likewise, there was no dark period under LL condition, but no photooxidative stress was observed in lettuce. Thus, it is concluded that photooxidative stress induced by CL can be attributed to excessive daily light integral instead of circadian asynchrony.

## Introduction

Continuous light (CL) is a particular lighting pattern that prolongs lighting duration to the utmost extent and can be applied in protected horticulture, especially in the plant factory with artificial light. Previous studies have shown that CL has many positive effects on plants, but at the same time, more studies have focused on the adverse effects of CL (Sysoeva et al., 2010; Velez-Ramirez et al., 2011). Several lines of evidence suggest that CL-induced injury is strongly correlated with photooxidative pressure and the accumulation of reactive oxygen species (ROS) (Velez-Ramirez et al., 2011). Plant exposure to light conditions that saturate CO_2_ assimilation and produce excess excitation energy will result in the accumulation of ROS and thus induce photooxidative stress in photosynthetic cells (Haghjou et al., 2009). It has been proved that plant species with higher antioxidant contents (Demers and Gosselin, 2002) or greater ROS-detoxifying enzyme activities (Murage and Masuda, 1997) showed lower or no CL-induced injury compared with other plant species. In our previous study on lettuce (Zha et al., 2019), ROS contents and antioxidant enzyme activities were distinctly elevated by CL compared with those under a normal photoperiod (16/8 h), although no leaf injury was observed under CL.

Plants have developed a complex antioxidant defense system to overcome oxidative stress. The system consists of two components: enzymatic and non-enzymatic antioxidants (Apel and Hirt, 2004; Gill and Tuteja, 2010; Soares et al., 2018). Enzymatic antioxidants are the enzymes involved in the ROS-scavenging pathway, viz. catalase (CAT), superoxide dismutase (SOD), ascorbate peroxidase (APX), dehydroascorbate reductase (DHAR), monodehydroascorbate reductase (MDHAR), and glutathione reductase (GR). Non-enzymatic antioxidants comprise several small-molecule antioxidants, such as carotenoids, ascorbate (AsA), tocopherol, and glutathione (GSH) (Apel and Hirt, 2004; Gill and Tuteja, 2010; Soares et al., 2018). Among them, AsA is the most abundant and powerful antioxidant in scavenging ROS, as it can donate electrons in a number of enzymatic and non-enzymatic reactions (Gill and Tuteja, 2010). The crucial role of AsA in antioxidant defense and stress protection has been well established (Smirnoff and Pallanca, 1996; Apel and Hirt, 2004; Bartoli et al., 2004). In addition to scavenging ROS, AsA has multiple other functions in photoprotection, including regeneration of α-tocopherol from α-tocopheryl radicals, acting as a cofactor for violaxanthin de-epoxidase and donation of electrons to photosystem II (Smirnoff and Wheeler, 2000). The hypersensitivity of ascorbate-deficient *Arabidopsis thaliana vtc* mutants to oxidative stresses also highlights the importance of AsA in the plant tolerance response to oxidative stresses (Smirnoff and Wheeler, 2000).

Moreover, in addition to AsA itself, AsA metabolism also occupies an extremely important role in the antioxidant defense system, as the majority of enzymatic (APX, MDHAR, DHAR, and GR) and non-enzymatic (AsA and GSH) antioxidants participate in AsA metabolism (Ntagkas et al., 2018). The AsA recycling pathway (i.e., the AsA-GSH cycle) is one of the central and most efficient antioxidant systems for removing ROS and maintaining the cellular redox state (Apel and Hirt, 2004; Bartoli et al., 2006). During the cycle, AsA is oxidized to monodehydroascorbate (MDHA) by APX and accomplish with the detoxification of hydrogen peroxide (H_2_O_2_). MDHA can be reduced back to AsA by MDHAR or re-reduced to dehydroascorbate (DHA) by spontaneous dismutation (Buettner and Jurkiewicz, 1996). DHA is enzymatically reduced by DHAR using an electron provided by GSH, which is then oxidized to oxidized glutathione (GSSG) and subsequently regenerated by GR (Potters et al., 2004). Moreover, AsA biosynthesis is also connected with ROS metabolism through L-galactono-1,4-lactone dehydrogenase (GalLDH), which catalyzes the final step of AsA biosynthesis in mitochondria. Complexes I and III of the mitochondrial electron transport chain are the main sites of O_2_^•−^ production (Gill and Tuteja, 2010). Recent research has suggested that GalLDH is an assembly factor of complex I, but this function is independent of its role in AsA synthesis (Schimmeyer et al., 2016; Zhang et al., 2016). However, Schimmeyer et al. (2016) also pointed out that a fraction of GalLDH is not associated with complex I intermediate. In addition, it is well recognized that GalLDH delivers electrons to cytochrome c, which is a removable electron transporter between complexes III and IV (Bartoli et al., 2000; Blokhina and Fagerstedt, 2010). Thus it can be speculated that at least the part of GalLDH that catalyzes AsA biosynthesis is involved in O_2_^•−^ production *via* cytochrome c. Considering the powerful antioxidant features of AsA metabolism and its tight connection to ROS metabolism, it is essential to explore the responses of AsA metabolism to light stresses, especially CL, which has been poorly investigated.

Light intensity has been proven to have a great influence on AsA level (Smirnoff and Pallanca, 1996). Within a certain range of light intensity, the stronger the irradiance intensity is, the higher the AsA contents in leaves (Bartoli et al., 2006; Dowdle et al., 2007; Fukunaga et al., 2010). The AsA pool size is comprehensively regulated by all enzymes involved in its metabolism, and each of them has been shown to present an obvious response to light intensity in previous studies (Smirnoff and Pallanca, 1996). For instance, growth at low light has been found to decrease the transcript level of GalLDH (Tabata et al., 2002). An increase in MDHAR and DHAR activities in response to high irradiance was found in tobacco (Chen and Gallie, 2004) and *Arabidopsis* (Bartoli et al., 2006). Although the response of each enzyme to light intensity has been widely investigated, it has not yet been concluded which enzyme is the most sensitive and plays the most important role in the response to light stress.

Light intensity also acts as a vital environmental factor that influences CL effects. For CL-sensitive plant species (e.g., tomato), CL-induced injury is more severe at higher light intensity (Murage et al., 1997). It has been proven that circadian asynchrony caused by constant light signaling seems to be the main factor provoking the CL-induced injury in tomato (Velez-Ramirez et al., 2017). Comparatively, lettuce seems to be more tolerant to CL than tomato. Although lettuce also had elevated ROS and antioxidant contents under CL at normal light intensity (200 µmol·m^−2^·s^−1^), it can grow normally and gain greater biomass without observable leaf injury according to our previous study (Zha et al., 2019). Thus, it is necessary to clarify the tolerance levels of lettuce to the intensity and duration of CL, which can be evaluated by oxidative stress, antioxidant capacity (ascorbate metabolism), and chlorophyll fluorescence responses of lettuce. In addition, investigating the oxidative stress responses of lettuce to CL under low light intensity could help us to ascertain whether circadian asynchrony will lead to stress in lettuce. Beyond these points, another aim of this study was to explore the differential responses of the enzymes involved in AsA metabolism to the light intensity of CL, thereby determining which enzyme pays the greatest contribution to resisting CL-induced stress. By studying and investigating of these problems, we could provide an optimal CL strategy for lettuce production in a plant factory and offer a reference for screening and breeding lettuce genotypes with higher antioxidant efficiency through the AsA metabolism.

## Materials and Methods

### Plant Materials and Light Treatments

The experimental trial was carried out in an environment-controlled plant factory at 23 ± 3 °C, HR 50–60%. Lettuce (*Lactuca sativa* L. cv. ‘Yidali’) seeds were germinated in the sponge under white LED light for 15 days and then transplanted into a recirculating hydroponic culture system equipped with red (R) and blue (B) LED lights with a light quality of 3R:1B, which was appropriate for lettuce growth according to previous research (Cope et al., 2014). To adapt to the new light quality and cultivation system, seedlings were acclimated for 10 days under a uniform light intensity and photoperiod after transplanting. The light intensity and photoperiod were 200 µmol·m^−2^·s^−1^ and 16/8 h, respectively, during the germination and acclimation stage (**Table 1**). After acclimation, seedlings were randomly divided into three groups (39 plants for each group) to be exposed to CL of different light intensities: low light (LL; 100 µmol·m^−2^·s^−1^), medium light (ML; 200 µmol·m^−2^·s^−1^), and high light (HL; 300 µmol·m^−2^·s^−1^) (**Table 1**). The composition of the applied nutrient solution was as follows (mM): 0.75 K_2_SO_4_, 0.5 KH_2_PO_4_, 0.1 KCl, 0.65 MgSO_4_·7H_2_O, 1.0 × 10^−3^ H_3_BO_3_, 1.0 × 10^−3^ MnSO_4_·H_2_O, 1.0 × 10^−4^ CuSO_4_·5H_2_O, 1.0 × 10^−3^ ZnSO_4_·7H_2_O, 0.1 EDTA-Fe, 5 × 10^−6^ (NH_4_)_6_Mo_7_O_24_·4H_2_O and 4.0 Ca(NO_3_)_2_·4H_2_O (pH: 5.8; EC: 1.38 mS·cm^−1^). The nutrient solution was recirculated for 60 min every day. Light was provided by red and blue light LED panels (50×50 cm, Shenzhen Huihao Optoelectronic Co. Ltd., Shenzhen, P. R. China) with the peak wavelengths of 660 and 430 nm. A light sensor logger (Li-1500, Lincoln, NE, USA) and a quantum sensor (LI-190R, Lincoln, Nebraska, USA) were used to measure light intensity at the canopy level.

**Table 1:** Light environmental conditions (light quality, photoperiod, and light intensity) of each treatment at each growth stage of lettuce.

Treatments	Germination stage (15 days)	Acclimation stage (10 days)	Treatment stage (12 days)
LL	W, 16/8 h	3R:1B, 16/8 h	3R:1B, 24/0 h, 100 μmol·m^−2^·s^−1^
ML	200 μmol·m^−2^·s^−1^	200 μmol·m^−2^·s^−1^	3R:1B, 24/0 h, 200 μmol·m^−2^·s^−1^
HL			3R:1B, 24/0 h, 300 μmol·m^−2^·s^−1^

W: white LED light, 3R:1B: red and blue LED light with a ratio of 3:1.

### Sampling and Measurements of Growth Parameters

Four lettuce plants of each treatment were sampled at 21:00 (the end of the light period at germination and acclimation stages) on days 0, 3, 6, 9, and 12 after the start of the treatments and remained as four biological replicates for the physiological determination. Three to five fully expanded leaves without petioles were collected from each plant and mixed as one biological replicate. The collected leaves were immediately wrapped with aluminum foil and frozen in liquid nitrogen and then stored at −80 °C until analysis. On the 12th day after treatment, another five plants were sampled to measure the fresh weight (FW) of shoots and roots, leaf area, leaf FW, and dry weight (DW) of shoots and roots. After removing the petiole, the remaining mesophyll part was used to determine the leaf FW and leaf area, and the specific leaf FW was then calculated as the ratio of leaf FW to leaf area of the same plant. The leaf area was determined by an area meter (LI-3100, Li-Cor Biosciences, Lincoln, Nebraska, USA). The DW of shoots and roots was weighed after drying at 80 °C for 48 h, and used to calculate the shoot DW/FW and root/shoot ratio.

### Measurements of Superoxide Anion, Hydrogen Peroxide, and Malondialdehyde Contents

The hydrogen peroxide (H_2_O_2_) content was determined according to the method of Brennan and Frenke (1977). 0.1 g fresh frozen leaf tissue was homogenized in 1 mL pre-cooled acetone, and then centrifuged at 10,000g for 20 min at 4 °C. After centrifugation, 0.1 mL of 10% (v/v) titanium sulfate and 0.2 mL ammonia were added to the 1 mL of extract and the mixture was centrifuged at 4,000g for 10 min at 25 °C. The precipitation was dissolved in 1 mL 2 M H_2_SO_4_ to measure the absorbance at 412 nm with a UV–VIS spectrophotometer (Shimadzu UV-1800, Kyoto, Japan).

The superoxide anion (O_2_^•−^) content was determined according to the method reported by Elstner and Heupel (1976) with some modification. 0.1 g frozen leaf tissue was homogenized in 1 mL of 50 mM phosphate buffer solution (PBS, pH 7.8) which contained 1 mM EDTA, 0.3% (v/v) Triton X-100, and 2% (w/v) polyvinylpyrrolidone (PVP). The homogenate was centrifuged at 10,000g for 20 min at 4 °C. 0.5 mL supernatant was mixed with 0.4 mL 1 mM hydroxylamine hydrochloride and then incubated at 37 °C for 20 min. After incubation, 0.3 mL 17 mM 1,4-aminobenzenesulfonic acid and 0.3 mL 7 mM α-naphthylamine were added into the mixture and incubated at 37 °C for 20 min again. Subsequently, 0.5 mL chloroform were added to the reaction, mixed, and then centrifuged at 8,000g for 5 min at 25 °C. Finally, 1 mL of upper aqueous phase was collected to measure the absorbance at 530 nm.

The malondialdehyde (MDA) content was determined using the method described by Yang et al. (2010). 0.1 g frozen leaf tissue was homogenized in 1 mL cold 10% trichloroacetic acid (TCA), and centrifuged at 15,000g for 10 min at 4 °C. 0.5 mL supernatant and 0.5 mL 0.6% thiobarbituric acid were mixed and boiled at 100 °C for 20 min, and then quickly cooled to room temperature. The supernatants were collected for measurement after centrifuge the reaction mixture at 15,000g for 10 min. The absorbance monitored at 450, 532 and 600 nm was used to calculate the MDA content.

### Non-Enzymatic Antioxidants Assays

Determination of total ascorbate (T-AsA) and AsA was carried out according to Gillespie and Ainsworth (2007). 0.1 g frozen leaf tissue was homogenized in 1 mL cold 6% (w/v) TCA and then centrifuged at 13,000g at 4 °C for 15 min. The supernatant was collected for detecting T-AsA and AsA contents. For T-AsA determination, 0.4 mL supernatant was mixed with 0.2 mL 75 mM PBS (pH 7.4) and 0.2 mL 10 mM DTT and then incubated for 10 min at 25 °C to reduce all DHA to AsA. After incubation, 0.2 mL of 0.5% (w/v) N-ethylmaleimide (NEM) was added to remove excess DTT. Afterwards 1 mL 10% (w/v) TCA, 0.8 mL 43% H_3_PO_4_, 0.8 mL 4% (w/v) α-α’-bipyridyl in 70% ethanol, and 0.4 mL 3% (w/v) FeCl_3_ were successively added into reaction mixture. The reaction mixtures were then incubated in water bath at 37 °C for 1 h and quantified at 525 nm. AsA was analyzed in a similar manner except that 0.4 mL deionized H_2_O was substituted for DTT and NEM. DHA content was calculated as the difference between the contents T-AsA and AsA.

GSH and GSSG were determined by a method adapted from Rahman et al. (2007). 0.1 g leafy tissue was homogenized in 1 mL cold 5% (w/v) sulfosalicylic acid and then centrifuged at 13,000g for 10 min at 4 °C. For analyses of T-GSH and GSSG contents the supernatant was collected. To assay T-GSH content, 50 µL deionized H_2_O, 730 µL 50 mM PBS (pH 7.5) containing 2.5 mM ethylenediaminetetraacetic acid (EDTA), 80 µL 12.5 mM 5,5’-dithiobis-2-nitrobenzoic acid (DTNB), and 20 µL 10 mM NADPH were added into 100 µL supernatant in turn. The reaction mixture was then incubated at 25 °C for 10 min. After incubation, 20 µL 50 U·mL^−1^ GR were added to the mixture and quantified at 412 nm immediately after 3 min reaction. GSSG content was analyzed in a similar manner except that the supernatant was incubated with 50 µL 10% (v/v) 2-vinylpyridine instead of deionized H_2_O at 25 °C for 1 h to remove GSH. GSH content was calculated as the difference between the contents of T-GSH and GSSG.

### Enzymatic Antioxidants Assays

GalLDH (EC 1.3.2.3) activity was determined according to the method of Li et al. (2009). 0.3 g fresh frozen leaf tissue was homogenized in 2 mL of the following extraction media: 100 mM PBS (pH 7.4), 0.4 M sucrose, 10% (v/v) glycerol, 1 mM EDTA, 0.3% (v/v) mercaptoethanol, and 1% (w/v) PVP. The reaction mixture (1 mL) contained 50 mM PBS (pH 7.8), 1.05 mg·mL^−1^ Cyt c, 5.6 mM L-Galactono-1,4-lactone, and 0.1 mL enzyme extract. The reaction was triggered by L-Galactono-1,4-lactone and the absorbance at 550 nm was monitored. One unit of the enzyme was defined as the amount that oxidized 1 mmol of L-galactono-1,4-lactone corresponding to the reduction of 1 µmol of Cyt c per min.

For the APX (EC 1.11.1.11) activity assay, 0.3 g fresh frozen leaf tissue was extracted in 2 mL 100 mM PBS (pH 7.0) which contained 0.2 mM EDTA and 1 mM AsA according to Nakano and Asada (1981). The reaction mixture, contained 50 mM PBS (pH 7.8), 0.5 mM AsA, 0.25 mM H_2_O_2_ and 0.1 mL of the extract. The reaction was started by adding H_2_O_2_. The activity of APX was measured following the decrease in the absorbance at 290 nm. One unit of the enzyme was defined as the amount that oxidized 1 mmol of AsA per min.

For the assays of MDHAR (EC 1.6.5.4), DHAR (EC 1.8.5.1), and GR (EC 1.8.1.7) activities, 0.3 g fresh frozen leaf tissue was homogenized in 2 mL of the following pre-cooled extraction media: 50 mM PBS (pH 7.5), 1 mM EDTA, 1 mM DTT, 0.1% (v/v) Triton X-100, 0.2% (v/v) mercaptoethanol, and 2% (w/v) PVP. The resulting slurry was centrifuged at 13,000g for 10 min at 4 °C. The supernatants were collected and used for the assays of enzyme activities according to Ma and Cheng (2004). The reaction mixture (3 mL) for MDHAR assay consisted of 50 mM Hepes-KOH (pH 7.6), 0.5 mM ascorbate, 0.3 mM NADH, 0.5 units AsA oxidase (AO), and 0.1 mL enzyme extract. The reaction was triggered by AO and the absorbance at 340 nm was monitored. One unit of the enzyme was defined as the amount that oxidized 1 mmol of NADH per min. The reaction mixture (3 mL) for DHAR assay contained 100 mM Hepes-KOH (pH 7.0), 1 mM EDTA, 2.5 mM GSH, 0.6 mM DHA, and 0.1 mL enzyme extract. The reaction was triggered by DHA and the absorbance at 265 nm was monitored. One unit of the enzyme was defined as the amount that reduced 1 mmol of DHA per min. The reaction mixture (3 mL) for GR assay contained 100 mM Tris–HCl (pH 8.0), 1 mM EDTA, 3 mM GSSG, 0.6 mM NADPH, and 0.1 mL enzyme extract. The reaction was triggered by NADPH and the absorbance at 340 nm was monitored. One unit of the enzyme was defined as the amount that oxidized 1 mmol of NADPH per min.

### Chlorophyll Fluorescence Measurements

Chlorophyll fluorescence parameters of the lettuce leaves were measured using a Pulse Amplitude Modulation Fluorometer (MINI-PAM, Heinz Walz, Germany). The second fully expanded leaves of six plants from each treatment were measured on the 6th and 12th days after treatment. The values of minimal fluorescence (*F*_o_) and maximal fluorescence (*F*_m_) were determined after leaves were dark-adapted for 30 min with leaf clips. Subsequently, the leaves were then light-adapted for 30 min to measure steady-state yield of fluorescence in the light (*F*_s_). The maximum PSII quantum efficiency (*F*_v_/*F*_m_), effective quantum yield of PSII (ΦPSII), the coefficient of photochemical quenching (qP) and non-photochemical quenching (qN) were calculated as follows: *F*_v_/*F*_m_ = (*F*_m_−*F*_o_)/*F*_m_; ΦPSII = (*F*_m_’−*F*_s_)/*F*_m_’; qP = (*F*_m_’−*F*_s_)/(*F*_m_’−*F*_o_’); and qN = 1−(*F*_m_’−*F*_o_’)/(*F*_m_−*F*_o_); *F*_o_’ = *F*_o_/(*F*_v_/*F*_m_ + *F*_o_/*F*_m_’).

### Statistical Analysis

Significant differences between different light intensity treatments at each time point were tested by Tukey’s test at 95% confidence. The effects of two factors, ‘light intensity (PPFD)’ and ‘days’, and their interaction were tested by two-way analysis of variance (ANOVA). Correlation and significance tests among physiological parameters were calculated using the Pearson correlation coefficient with a two-tailed test. These analyses were performed in the statistical software SPSS 18.0 (International Business Machines Corporation). In addition, a principal component analysis (PCA) performed in Canoco 5.0 (Microcomputer Power, Ithaca, NY, USA) was applied to examine how physiological parameters changed according to the light intensity of CL on each sampling day.

## Results

### Plant Growth

The growth of lettuce plants under CL for 12 days presented marked responses to light intensity (**Table 2**). The shoot FW, shoot DW, shoot DW/FW ratio, root/shoot ratio, and specific leaf FW progressively increased with light intensity, while there was no significant effect of light intensity on leaf area under CL. Differences in growth parameters, including shoot FW, shoot DW, shoot DW/FW ratio, and specific leaf FW, between LL-grown plants and ML-grown plants were greater than those between ML- and HL- grown plants. In contrast, the difference in the root/shoot ratio was much greater between ML- and HL-grown plants than between LL- and ML-grown plants.

**Table 2:** The shoot fresh weight (FW), shoot dry weight (DW), shoot DW/FW, root/shoot ratio, leaf area, and specific leaf FW of lettuce plants grown under continuous light of different light intensities: low light (LL, 100 μmol·m^−^^2^·s^−^^1^), medium light (ML, 200 μmol·m^−^^2^·s^−^^1^), and high light (HL, 300 μmol·m^−^^2^·s^−^^1^).

Light intensity	Shoot FW (g)	Shoot DW (g)	Shoot DW/FW (%)	Root/shoot ratio	Leaf area (dm^2^)	Specific leaf FW (g/dm^2^)
LL	76.9 c	2.06 b	2.62 c	0.12 b	12.12 a	3.31 c
ML	98.8 b	3.48 a	3.38 b	0.14 b	12.84 a	4.15 b
HL	109.9 a	4.16 a	3.71 a	0.18 a	11.96 a	4.87 a

The samples were taken at 12th day after treatment. Values are means of five replicates. Different letters indicate significant differences between different light intensity treatments at p < 0.05 according to Tukey test.

### Leaf Oxidative Stress

**Figure 1:**
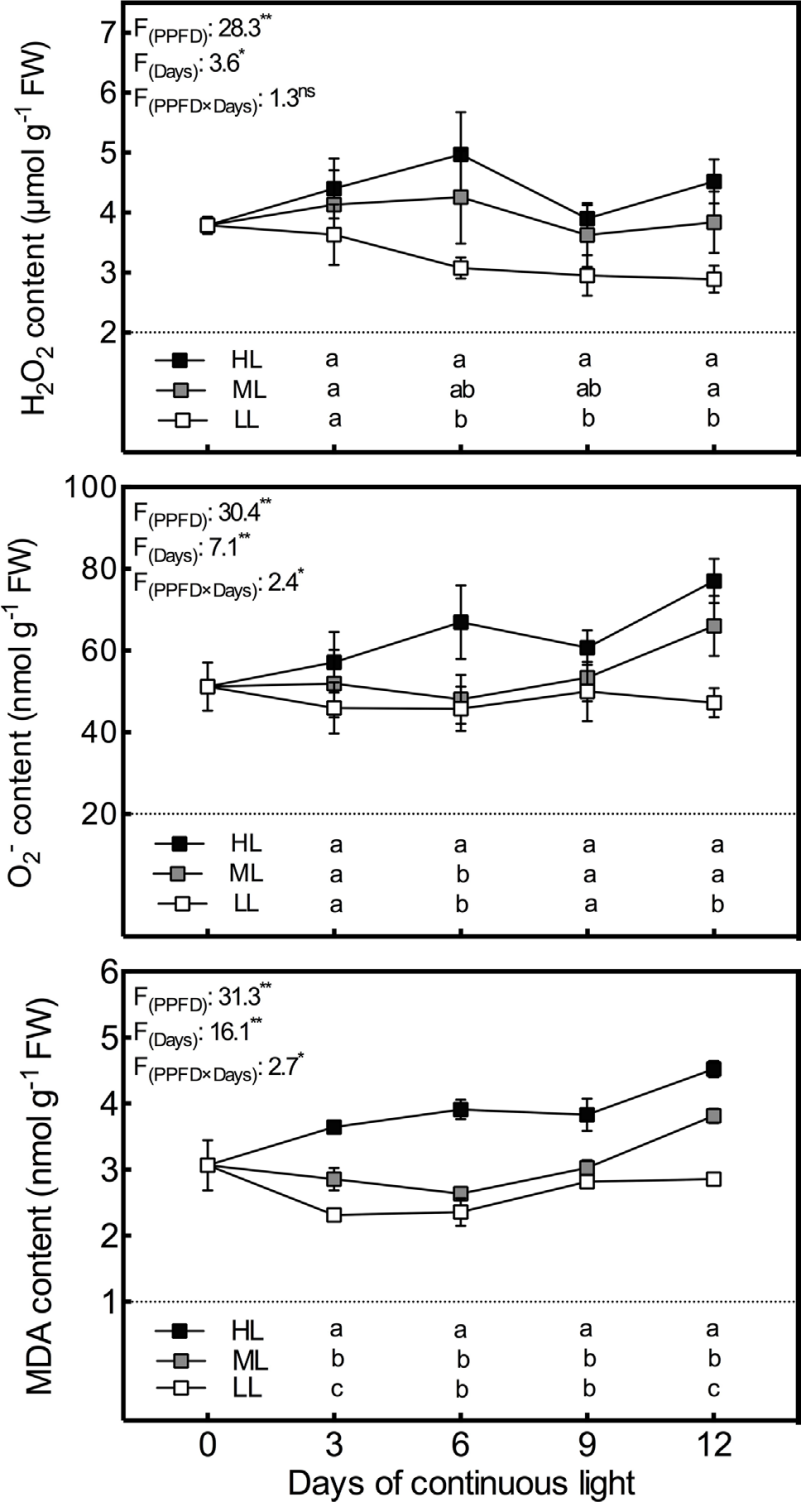
Changes in H_2_O_2_, O_2_^•−^, and MDA contents in lettuce leaves grown under continuous light of different intensities: low light (LL, 100 μmol·m^−2^·s^−1^), medium light (ML, 200 μmol·m^−2^·s^−1^), and high light (HL, 300 μmol·m^−2^·s^−1^). Values are means of four replicates ± SD. Different letters indicate significant differences between different light intensity treatments at *p* < 0.05 according to Tukey test. F values and significance of the two-way ANOVA considering the factors light intensity (PPFD), days and their interactions were given in the inset. ^ns^, * and ** indicate nonsignificant and significant at *p* < 0.05 and 0.01 respectively.

In order to investigate photooxidative stress induced by CL of different intensities, H_2_O_2_ and O_2_^•−^ levels in lettuce leaves were examined. As shown in the **Figure 1**, H_2_O_2_ and O_2_^•−^ contents had a positive correlation with light intensity at all time points. The variation trends in H_2_O_2_ levels in HL and ML leaves and in O_2_^•−^ levels in HL leaves were much the same. These levels increased during the first 6 days, declined on day 9 and increased again on the last day, with the most pronounced variation seen in the HL plants. H_2_O_2_ and O_2_^•−^ contents in LL leaves dwindled and remained at a relatively stable level, respectively, during the experiment. O_2_^•−^ contents in ML leaves remained stable during the first 9 days, as seen in LL leaves, but exhibited a sharp increase on day 12, as seen in HL leaves. The lipid peroxidation degree was determined in terms of MDA levels to examine ROS induced oxidative damage in lettuce. The MDA content had almost an identical variation tendency to O_2_^•−^, but the differences between the light intensity treatments were more significant for MDA content (**Figure 1**).

### Ascorbate Pool

**Figure 2:**
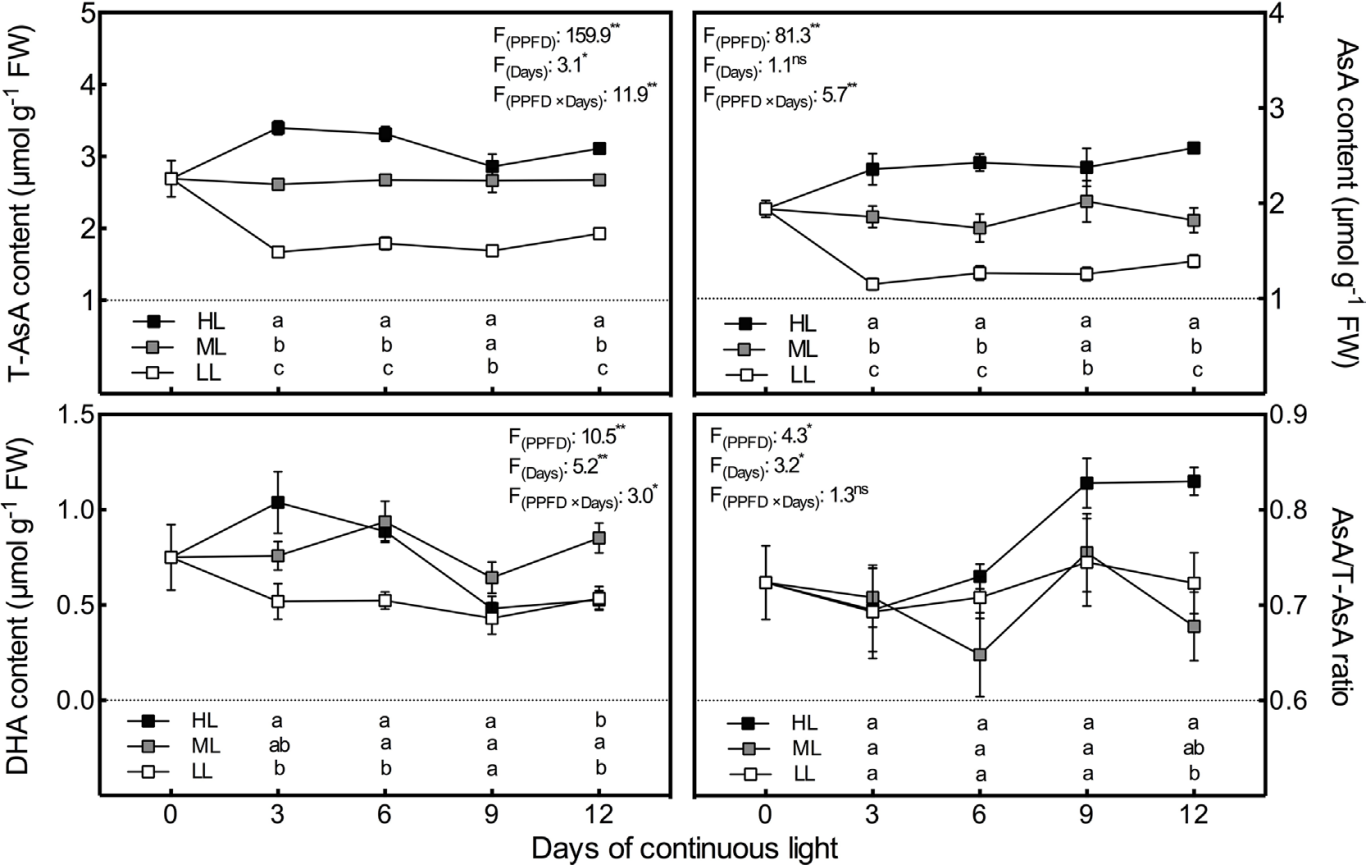
Changes in total ascorbate (T-AsA), ascorbate (AsA) and dehydroascorbate (DHA) contents as well as AsA/T-AsA ratio in lettuce leaves grown under continuous light of different intensities: low light (LL, 100 μmol·m^−2^·s^−1^), medium light (ML, 200 μmol·m^−2^·s^−1^), and high light (HL, 300 μmol·m^−2^·s^−1^). Values are means of four replicates ± SD. Different letters indicate significant differences between different light intensity treatments at *p* < 0.05 according to Tukey test. F values and significance of the two-way ANOVA considering the factors light intensity (PPFD), days and their interactions were given in the inset. ^ns^, * and ** indicate nonsignificant and significant at *p* < 0.05 and 0.01 respectively.

The chang tendencies of T-AsA and AsA levels with time and with light intensity were quite similar (**Figure 2**). Both had significant positive correlations with light intensity at all time points and remained at relatively stable levels under the same light intensity. However, the increasing trend of DHA content with light intensity was only observed on day 3 (**Figure 2**). After day 3, DHA levels decreased substantially and constantly under HL condition, which resulted in a synchronous increase in the AsA/T-AsA ratio. By day 12, the DHA level and the AsA/T-AsA ratio in HL leaves emerged as the lowest and highest, respectively, among all treatments.

### Glutathione Pool

**Figure 3:**
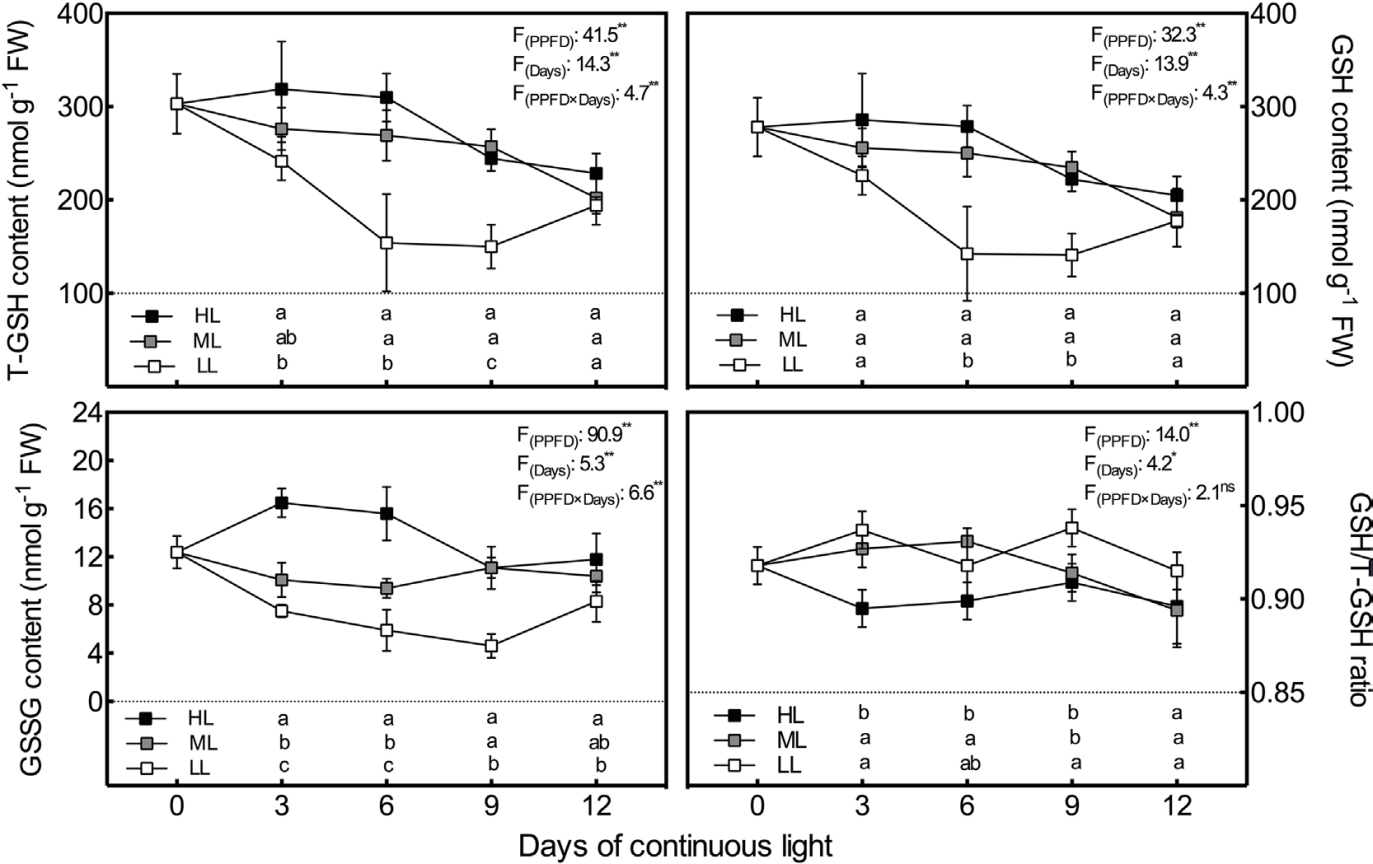
Changes in total glutathione (T-GSH), glutathione (GSH) and oxidized glutathione (GSSG) contents as well as GSH/T-GSH ratio in lettuce leaves grown under continuous light of different intensities: low light (LL, 100 μmol·m^−2^·s^−1^), medium light (ML, 200 μmol·m^−2^·s^−1^), and high light (HL, 300 μmol·m^−2^·s^−1^). Values are means of four replicates ± SD. Different letters indicate significant differences between different light intensity treatments at *p* 0.05 according to Tukey test. F values and significance of the two-way ANOVA considering the factors light intensity (PPFD), days and their interactions were given in the inset. ^ns^, * and ** indicate nonsignificant and significant at *p* 0.05 and 0.01 respectively.

Consistent with the AsA pool, the T-GSH contents had almost the same variation tendency as the GSH contents (**Figure 3**). In general, the T-GSH and GSH levels of all treatments presented a downward tendency during the experiment, but the tendency reversed during the last 3 days in LL leaves, leading to no difference among treatments on day 12. The T-GSH and GSH contents were markedly increased by the increase in CL light intensity on day 3 and day 6, and presented notably lower levels in LL leaves than in ML and HL leaves on days 6 and 9. Unlike GSH and T-GSH, GSSG contents were positively associated with light intensity during the whole experiment except on day 9, when there was no difference in GSSG between ML and HL leaves (**Figure 3**). Additionally, HL leaves had strikingly higher GSSG levels than ML and LL leaves on days 3 and 6, which led to a significantly lower GSH/T-GSH ratio in HL leaves on the same days.

### Enzyme Activity

**Figure 4:**
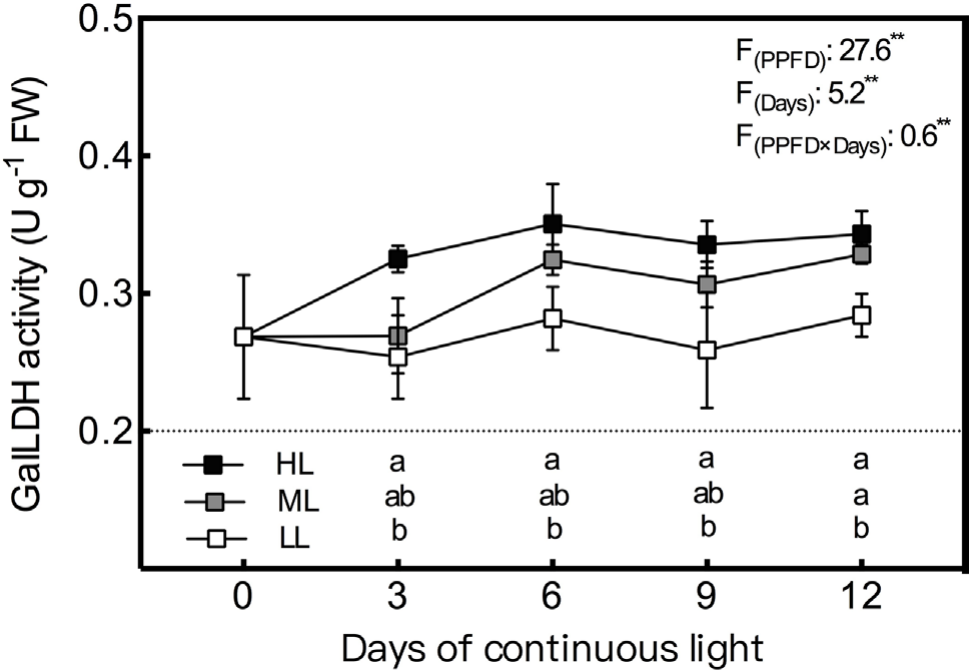
Changes in L-galactono-1,4-lactone dehydrogenase (GalLDH) activity in lettuce leaves grown under continuous light of different intensities: low light (LL, 100 μmol·m^−2^·s^−1^), medium light (ML, 200 μmol·m^−2^·s^−1^), and high light (HL, 300 μmol·m^−2^·s^−1^). Values are means of four replicates ± SD. Different letters indicate significant differences between different light intensity treatments at *p* < 0.05 according to Tukey test. F values and significance of the two-way ANOVA considering the factors light intensity (PPFD), days and their interactions were given in the inset. ** indicate nonsignificant and significant at *p* < 0.05 and 0.01 respectively.

**Figure 5:**
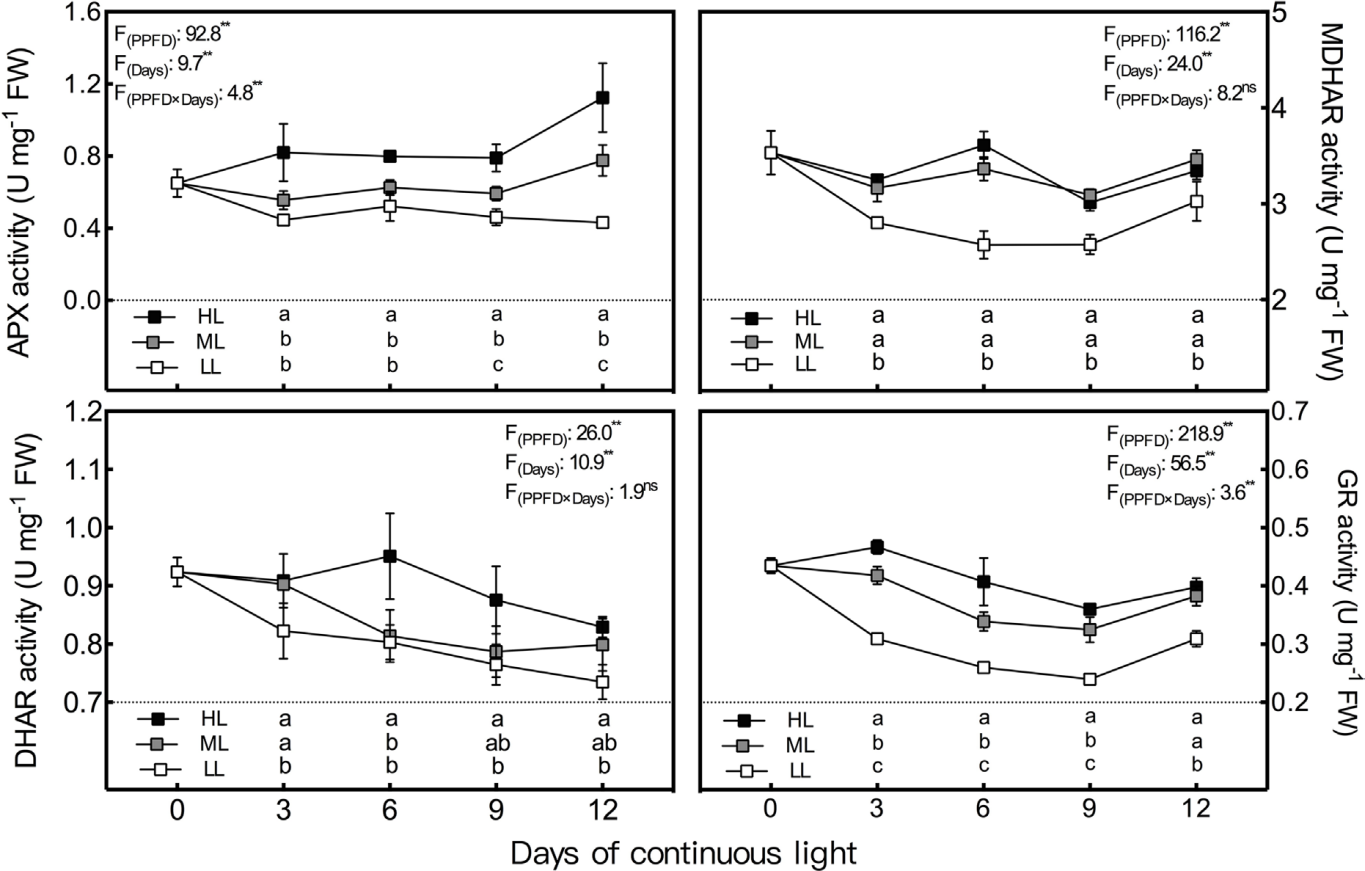
Changes in ascorbate peroxidase (APX), dehydroascorbate reductase (DHAR), monodehydroascorbate reductase (MDHAR) and glutathione reductase (GR) activities in lettuce leaves grown under continuous light of different intensities: low light (LL, 100 μmol·m^−2^·s^−1^), medium light (ML, 200 μmol·m^−2^·s^−1^), and high light (HL, 300 μmol·m^−2^·s^−1^). Values are means of four replicates ± SD. Different letters indicate significant differences between different light intensity treatments at *p* < 0.05 according to Tukey test. F values and significance of the two-way ANOVA considering the factors light intensity (PPFD), days and their interactions were given in the inset. ^ns^ and ** indicate nonsignificant and significant at *p* < 0.05 and 0.01 respectively.

During the whole experiment period, the GalLDH activity of lettuce leaves grown under CL remained the lowest and the highest in LL and HL leaves, respectively, and the difference between LL and HL leaves reached a significant level (**Figure 4**). In general, GalLDH activity in HL and LL leaves showed relatively stable levels under CL, while that of ML leaves presented an increasing trend from day 3 to day 6. Both APX and GR activities were progressively elevated by increasing light intensity (**Figure 5**); the former was more sensitive to HL condition, while the latter responded more notably to LL treatment. The APX activity remained steady in LL leaves but increased abruptly from day 9 to day 12 under ML and HL conditions. The GR activity of the three treatments had consistent variations with time; it decreased from day 3 to day 9, and then increased from day 9 to day 12. The MDHAR activity of LL leaves had a similar tendency to that of the GR activity and was dramatically lower than those of ML and HL leaves, which had almost identical levels of MDHAR activity (**Figure 5**). The DHAR activity of ML and LL leaves decreased gradually with time, and differences between them were not significant except on day 3. Under HL condition, DHAR activity increased during the first 6 days and then decreased constantly (**Figure 5**).

### Chlorophyll Fluorescence Parameters

**Figure 6:**
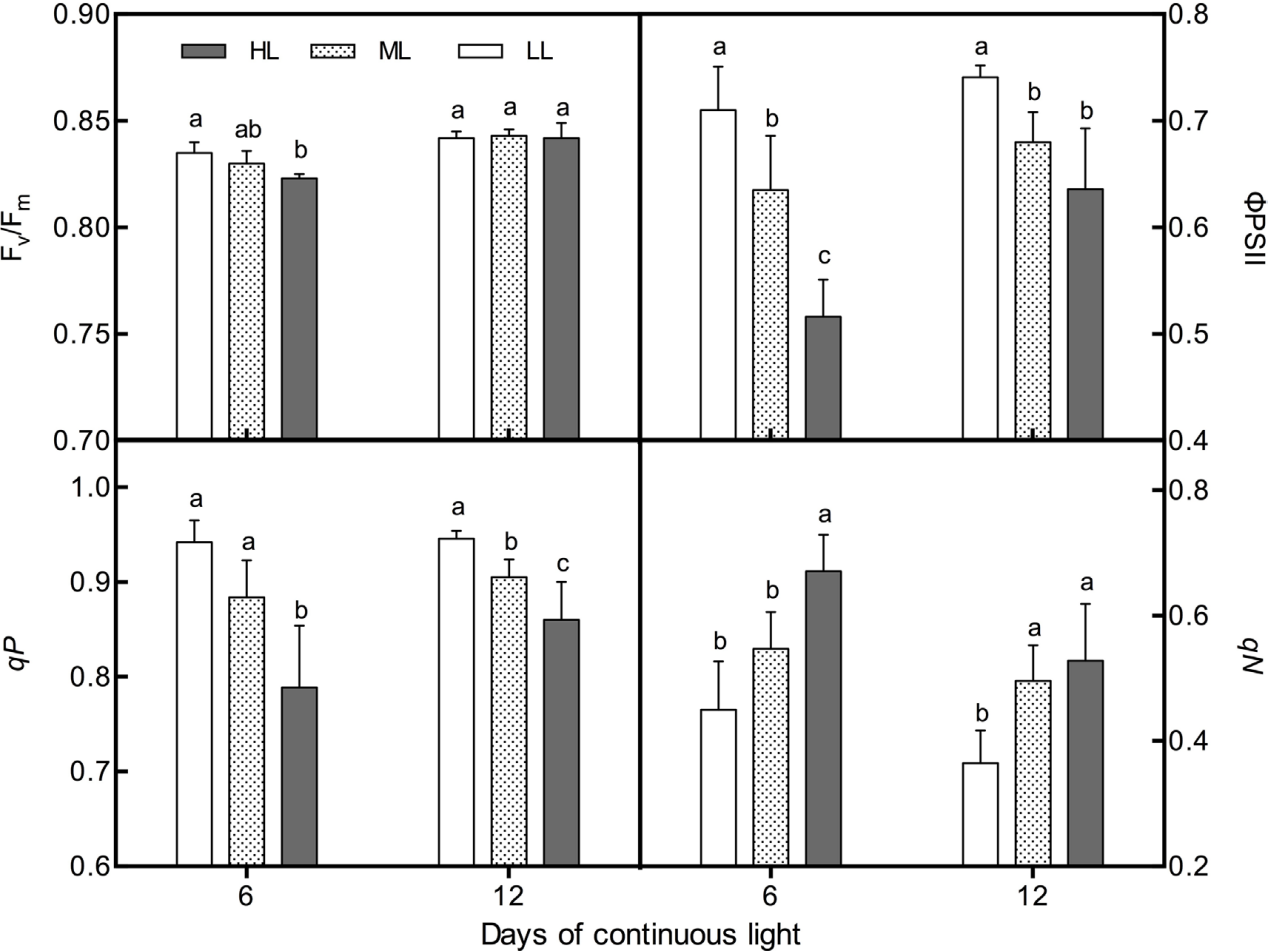
Changes in chlorophyll fluorescence parameters (*F*_v_/*F*_m_, qN, qP, and ΦPSII) in lettuce leaves grown under continuous light of different intensities: low light (LL, 100 μmol·m^−2^·s^−1^), medium light (ML, 200 μmol·m^−2^·s^−1^), and high light (HL, 300 μmol·m^−2^·s^−1^). Values are means of four replicates ± SD. Different letters indicate significant differences between different light intensity treatments at *p* < 0.05 according to Tukey test.

Under CL, the *F*_v_/*F*_m_ value had a negative correlation with light intensity on day 6. *F*_v_/*F*_m_ in HL leaves, with a value of 0.823, was significantly lower than that in LL leaves (**Figure 6**). There was no difference in *F*_v_/*F*_m_ among treatments on day 12. ФPSII and qP presented notably negative correlations with light intensity on both day 6 and day 12. However, qN remained positively associated with light intensity during the whole experiment (**Figure 6**). The values of *F*_v_/*F*_m_, ФPSII, and qP of all treatments increased from day 6 to day 12, and the increasing rates developed with light intensity, while the qN showed an opposite trend.

### Correlation Analysis and Principal Components Analysis

**Figure 7:**
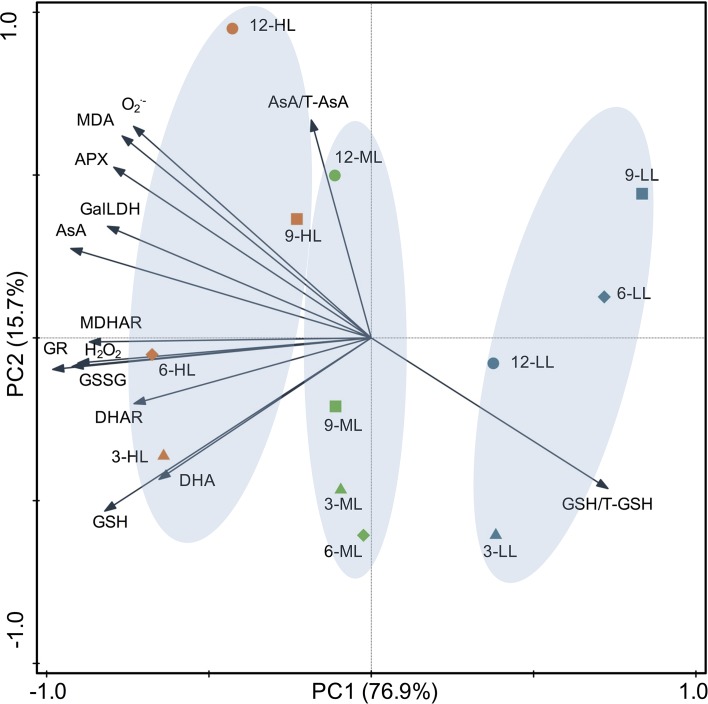
Principal component analysis of physiological parameters in lettuce leaves under continuous light of different intensities (yellow symbol-HL, 300 μmol·m^−2^·s^−1^; green symbol-ML, 200 μmol·m^−2^·s^−1^; blue symbol-LL, 100 μmol·m^−2^·s^−1^) at different days (△–day 3; ◇–day 6, □–day 9, ○–day 12). Biplot of the first two principal components (PC1,2) of the measured network topological properties. AsA, ascorbate; APX, ascorbate peroxidase; DHA, dehydroascorbate; DHAR dehydroascorbate reductase; GalLDH, L-galactono-1,4-lactone dehydrogenase; GR, glutathione reductase; GSH, glutathione; GSSG, oxidized glutathione; MDHA, monodehydroascorbate; MDHAR, monodehydroascorbate reductase; T-AsA, total ascorbate; T-GSH, total glutathione.

The correlation coefficients among the physiological parameters analyzed by Pearson’s correlation are listed in **Table 3**. Under CL, AsA showed significant correlations with all physiological parameters except DHA and AsA/T-AsA, which were negatively correlated with each other. Among the five enzymes involved in AsA metabolism, APX and DHAR had the greatest and least correlation with the AsA level, respectively. The GSSG level and GSH/T-GSH ratio had positive and negative correlations with the activities of all enzymes, respectively, while the GSH level only showed positive correlations with GR, DHAR, and MDHAR. H_2_O_2_ displayed positive correlations with all physiological parameters except for the redox ratios of the AsA and GSH pools (AsA/T-AsA and GSH/T-GSH). O_2_^•−^ and MDA had very strong correlations with each other, and both had strong correlations with APX and significant negative correlations with GSH/T-GSH. To obtain a more comprehensive understanding of the physiological responses of lettuce to different intensities of CL, the results of PCA were presented by a biplot (**Figure 7**). The first two principal components explained 76.9% and 15.7% of the data variability, respectively. Almost all physiological parameters, including AsA, DHA, GSH, GSSG, GalLDH, APX, MDHAR, DHAR, GR, H_2_O_2_, O_2_^•−^, and MDA, were negatively related to PC1, while the GSH/T-GSH ratio was positively related to PC1. The points of different light intensities were separated along PC1. Individuals occurring at sites under HL condition presented higher AsA and GSH pool sizes, greater enzyme activities, and higher ROS and MDA contents, but lower GSH/T-GSH ratios. Only the AsA/T-AsA ratio was positively related to PC2, which separated points of different sampling days under the same light intensity.

**Table 3:** Pearson’s correlation coefficients among physiological and biochemical parameters from lettuce exposed to continuous light of different intensities.

	ASA	DHA	AsA/ T-AsA	GSH	GSSG	GSH/ T-GSH	GR	DHAR	MDHAR	APX	GalLDH	H_2_O_2_	O_2_^·-^
AsA	–												
DHA	0.402	–											0.9–1.0
AsA/T-AsA	0.503	−0.583*	–										0.8–0.9
GSH	0.609*	0.706*	−0.135	–									0.7–0.8
GSSG	0.859**	0.695*	0.109	0.818**	–								0.6–0.7
GSH/T-GSH	−0.752**	−0.397	−0.288	−0.253	−0.749**	–							0.5–0.6
GR	0.786**	0.707*	0.022	0.783**	0.896**	−0.663*	–						
DHAR	0.647*	0.533	0.064	0.772**	0.742**	−0.363	0.750**	–					
MDHAR	0.715**	0.727**	−0.081	0.657*	0.787**	−0.642*	0.786**	0.498	–				
APX	0.881**	0.287	0.486	0.333	0.676*	−0.785**	0.671*	0.464	0.644*	–			
GalLDH	0.879**	0.487	0.306	0.446	0.775**	−0.817**	0.626*	0.468	0.769**	0.842**	–		
H_2_O_2_	0.806**	0.656*	0.065	0.810**	0.832**	−0.509	0.829**	0.819**	0.823**	0.735**	0.716**	–	
O_2_^·-^	0.817**	0.175	0.537	0.219	0.606*	−0.785**	0.596*	0.400	0.661*	0.928**	0.776**	0.660*	–
MDA	0.875**	0.212	0.572	0.270	0.687*	−0.841**	0.652*	0.417	0.656*	0.919**	0.829**	0.628*	0.965**

Each square indicates the Pearson’s correlation coefficient of a pair of parameters. Correlation was significant at *p < 0.05; **p < 0.01. The deeper the color is, the greater the correlation coefficient.

## Discussion

It is widely understood that the growth rate of plants increases with increasing light intensity in a certain range under a normal photoperiod (Kang et al., 2013). Our results indicated that this phenomenon also emerged under CL in lettuce with a range of light intensity from 100 to 300 µmol·m^−2^·s^−1^. Several growth parameters, including shoot FW, shoot DW, specific leaf FW, shoot DW/FW, and root/shoot ratio, increased with light intensity. In addition, when the light intensity increased from 100 to 200 µmol·m^−2^·s^−1^, the increments of these parameters, except the root/shoot ratio, were greater than those when the light intensity increased from 200 to 300 µmol·m^−2^·s^−1^. Thus, the positive effect of improving light intensity on lettuce growth under CL decreased when the light intensity exceeded 200 µmol·m^−2^·s^−1^. Unlike the above growth parameters, differences in leaf area were not observed under CL among different light intensities. Our previous study found that the increase in lettuce shoot biomass induced by CL was dependent on specific leaf FW rather than leaf area (Zha et al., 2019). This conclusion can also be applied to present study. In addition, consistent with specific leaf FW, leaf thickness was also increased by light intensity under CL as we observed (data was not shown). As leaves under CL were poor at retaining water (Arve et al., 2013), un-expanded leaf area and thicker leaves could help lettuce cope better with higher light intensity under CL by decreasing water loss and improving light utilization.

CL has been reported as a potential way to improve productivity in several studies (Sysoeva et al., 2010; Velez-Ramirez et al., 2011). However, under CL, even normal light intensity will lead to an excessive daily light integral (DLI), which might be a burden for plant. When the light energy received by the plant exceeds its own utilization capacity, excess light energy will lead to the accumulation of ROS by overstraining the reactions in chloroplasts during photosynthesis (Golan et al., 2006; Heyneke et al., 2013). It has been reported in our previous (Zha et al., 2019) and several other studies (Velez-Ramirez et al., 2011) that ROS content and ROS-detoxifying enzyme activity were upregulated by CL. As the duration of lighting is constant under CL, light intensity determines whether the DLI is excessive for plants. Our results revealed that the contents of O_2_·^−^ and H_2_O_2_ as well as MDA, the final product of lipid peroxidation, showed a positive correlation with light intensity under CL at each time point we investigated. These results are analogous to those obtained in previous studies, proving that high light intensity enhances the production and accumulation of ROS (Heyneke et al., 2013). The variations in ROS and MDA contents with time were inconsistent under different light intensity. ROS and MDA in ML and HL leaves presented considerable increases from day 9 to day 12, indicating that the antioxidant system might not be able to maintain the balance of ROS for more than 12 days under CL with high light intensity (>200 µmol·m^−2^·s^−1^). Nevertheless, LL leaves had stable O_2_·^−^ and MDA contents and dwindling H_2_O_2_ contents, demonstrating that no oxidative stress occurred under 100 µmol·m^−2^·s^−1^ CL, which had a lower DLI than normal light conditions (e.g., 200 µmol·m^−2^·s^−1^, 16/8 h) for lettuce growth. According to the study of Fu et al. (2012), with a photoperiod of 14/10 h, light stress did not appear until the light intensity exceeded 400 µmol·m^−2^·s^−1^. These results indicated that under 100–300 µmol·m^−2^·s^-1^ CL, the increase in the total DLI raised by light intensity leads to the oxidative stress in lettuce, rather than the light intensity itself. Additionally, differences between treatments and the changing tendency of MDA were very similar to those of O_2_·^−^ on the whole, leading to a strong correlation between them (P < 0.01, r = 0.959). This demonstrates that O_2_·^-^ is more responsible for lipid peroxidation than H_2_O_2_.

As the most abundant and powerful antioxidants for scavenging ROS, AsA and GSH function jointly in high-capacity redox-homeostatic H_2_O_2_-processing pathways (Mittler et al., 2004; Foyer and Noctor, 2011). According to the results, similar to ROS content, both AsA and GSH levels under CL were positively associated with light intensity. The results agree with those reported previously showing increments of AsA (Smirnoff and Wheeler, 2000) and GSH (Ogawa et al., 2004) in response to high light intensity in other plants. Compared to AsA, GSH presented greater sensitivity to LL conditions and smaller discrepancies between ML- and LL-grown leaves under CL (**Figures 2**, **3**), suggesting that low light intensity had greater effects on GSH than high light intensity. Similarly, it has been proposed that GSH is not as responsive as AsA to higher light intensity, although GSH can be elevated somewhat under high light intensity (Grace and Logan, 1996). Apart from the pool sizes of AsA and GSH, their redox ratios are also the key elements for efficient protection against the accumulation of ROS, as they play a substantial role in the maintenance of the cellular homeostasis and ROS signal transduction (Foyer and Noctor, 2011). It is shown here that DHA contents in lettuce leaves exposed to HL condition declined continuously from day 3 to day 9, with a consequent significantly higher AsA/T-AsA ratio on day 12. This caused a shift in the cell redox state toward the reduced form. However, significantly lower GSH/T-GSH ratios in HL leaves, which were induced by higher GSSG levels, were observed from day 3 to day 9. Furthermore, the correlation analysis demonstrates that O_2_·^−^ content was potentially related to the AsA/T-AsA ratio (*P* = 0.072, r = 0.537), but had a significant negative relation with the GSH/T-GSH ratio (*P* < 0.01, r = −0.785). Foyer and Noctor (2011) proposed that enhanced ROS availability has less impact on the AsA/T-AsA ratio than on the redox status of the GSH pool. The independence of AsA and GSH and/or the difference in redox potential between the AsA/DHA and GSH/GSSG couples might account for this phenomenon (Foyer and Noctor, 2011).

**Figure 8:**
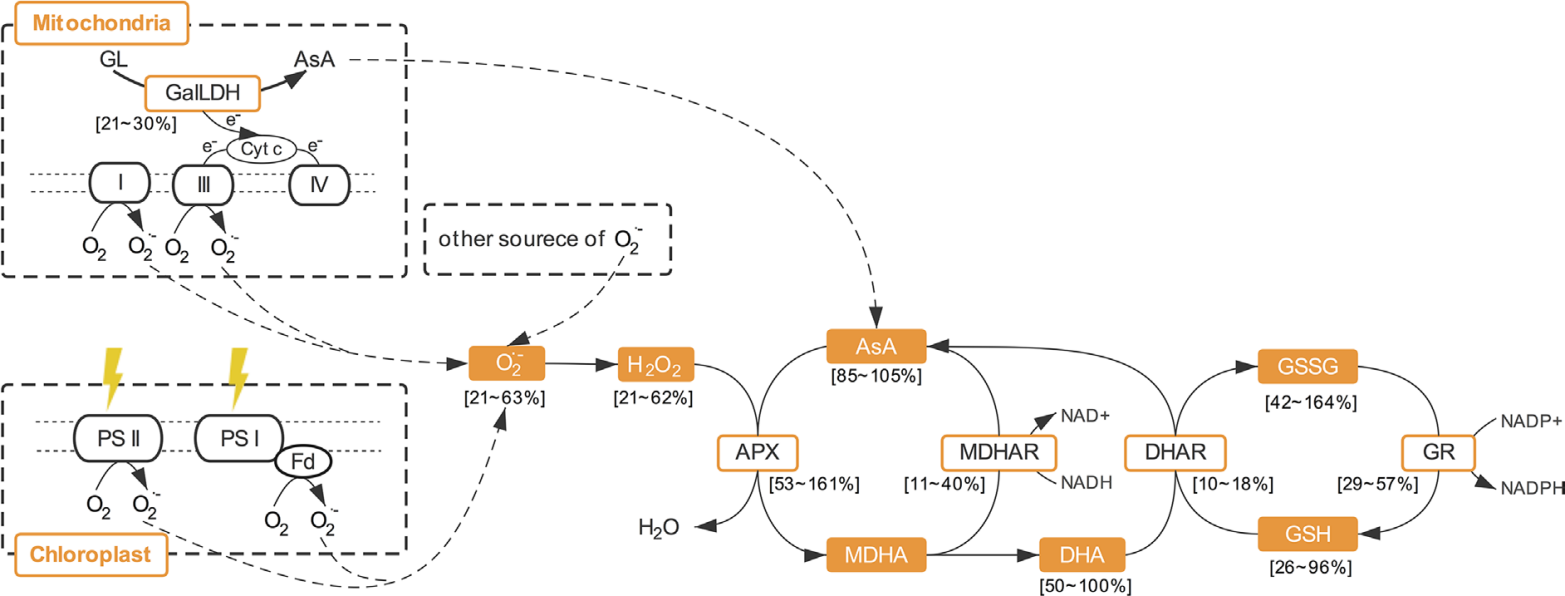
The roles of ascorbate metabolism in ROS scavenging. AsA, ascorbate; APX, ascorbate peroxidase; DHA, dehydroascorbate; DHAR dehydroascorbate reductase; GalLDH, L-galactono-1,4- lactone dehydrogenase; GR, glutathione reductase; GSH, glutathione; GSSG, oxidized glutathione; MDHA, monodehydroascorbate; MDHAR, monodehydroascorbate reductase; T-AsA, total ascorbate; T-GSH, total glutathione.

Through the AsA–GSH cycle, AsA content and its redox state can be maintained to resist ROS production (Gallie, 2013). Many authors have reported that the activities of enzymes involved in AsA regeneration (APX, MDHAR, DHAR, and GR) significantly increased in plants under various stress conditions (Haghjou et al., 2009; Singh et al., 2012; Gallie, 2013), and that the overexpression of the genes of these enzymes can improve the resistance of plants to various stresses (Chen et al., 2003; Gallie, 2013). In addition to the high light intensity which can cause oxidative stress, the activities or transcript levels of these enzymes were also elevated by increasing light intensity among the levels which are normal for plant growth (Karpinski et al., 1999; Chen and Gallie, 2004; Bartoli et al., 2006). As in previous research, the activities of all these four enzymes were enhanced by increasing light intensity of CL and presented significant positive correlation with ROS and MDA level in the present study. It is suggested that the promotion effects of light intensity on the activities of enzymes involved in AsA-GSH cycle under CL was partly because of the effect of light intensity itself and partly due to oxidative stress level which is decided by DLI. Moreover, susceptibilities of these enzymes to specific light intensities were different under CL in the present study. The discrepancies in APX and DHAR activities were greater between the ML and HL conditions, while greater differences in MDHAR and GR activities were observed between LL and ML. Among these enzymes, APX and DHAR presented the maximal and minimal responses to light intensity, respectively (**Figure 8**). Furthermore, APX had the strongest correlation with both H_2_O_2_ and O_2_·^−^ (**Table 3**). In the present study, the AsA synthesis capacity of CL-grown lettuce was also enhanced by higher light intensity, as seen in *Arabidopsis* grown under a normal photoperiod (Bartoli et al., 2006), demonstrated by greater GalLDH activity (**Figure 4**). Although increased GalLDH activity implies more electrons were delivered to cytochrome c, which might be a potential source of ROS generation (Bartoli et al., 2000; Blokhina and Fagerstedt, 2010), it made a greater contribution to AsA synthesis simultaneously.

Chlorophyll fluorescence can reflect the light absorption and utilization ability of PS II and can thereby be used to evaluate the photosynthetic capacity and abiotic stress degree of plants (Maxwell and Johnson, 2000). The data presented here showed that HL led to a significant decline in *F*_v_/*F*_m_, accompanied by lower ФPSII and qP on day 6. Reduction in *F*_v_/*F*_m_ indicated the occurrence of photoinhibition (Demmig et al., 1987; Dodd et al., 1998) and was due to the turndown of ФPSII (Porcar-Castell et al., 2014), which was attributed to reduced carbon fixation efficiency (Maxwell and Johnson, 2000) caused by HL-induced excess carbohydrate accumulation. The smaller qP values indicated a lower proportion of PSII reaction centers that were open (Maxwell and Johnson, 2000). As a result, light energy captured by the antenna pigments could not be effectively used to promote photosynthetic electron transport, resulting in excess energy dissipation in the form of heat, which leads to the rise of qN (Maxwell and Johnson, 2000) (**Figure 6**). Nevertheless, the increase in qN and the decrease in ФPSII often signifies that the photoinhibition is reversible (Demmig-Adams and Adams, 1992). Indeed, *F*_v_/*F*_m_ of ML and HL leaves rose back to the same level as LL leaves on day 12, and notable increases in ФPSII and qP, as well as a reduction in qN, were also observed in all treatments at the same time. These results imply that long-term CL could lead to an adaptive change in the photosynthetic apparatus to elevate light utilization ability. In addition, the greater ability of HL leaves to dissipate excess excitation energy should be attributed to higher contents of AsA, which functions as a cofactor for violaxanthin de-epoxidase and donates electrons to photosystem II (Smirnoff and Wheeler, 2000).

Under CL, both the energy and signaling components of light were continuously supplied to plants. However, there is no uniform conclusion about which components should be responsible for CL-induced injury. Velez-Ramirez et al. (2017) conjectured that circadian asynchrony caused by continuous light signaling was the main factor in CL-induced injury, because abnormal light/dark cycles (e.g., 6-h light/6-h dark or 24-h light/24-h dark) induce injury symptoms similar to those induced by CL on tomato (Velez-Ramirez et al., 2011; Velez-Ramirez et al., 2017). While in this study, although the circadian rhythms of all three treatments were disrupted by CL, LL condition did not evoke photooxidative stress in lettuce demonstrated by low ROS and MDA contents, normal chlorophyll fluorescence parameters, and healthy morphology without any injury symptoms. Moreover, all results indicated that stress level and antioxidant ability in lettuce positively relate to the light intensity. The light intensities we applied had little influence on oxidative stress under normal photoperiod according to previous reports (Fu et al., 2012), while differences of DLI corresponding to these light intensities were amplified by CL. The maximum DLI suggested for lettuce growth is 17 mol·m^−2^·d^−1^ (Brechner and Both, 2013), the DLI of LL and HL conditions were 8.64 mol·m^−2^·d^−1^and 25.92 mol·m^−2^·d^−1^, which were much lower and much higher than the suggested value, respectively. Hence, we can infer that CL-induced injury is mainly caused by excess DLI rather than circadian asynchrony, which means that energy components are more responsible for CL-induced injury than signaling components, at least in lettuce.

## Conclusions

In summary, the growth and physiological responses of lettuce plants to red and blue LED continuous light were highly associated with light intensity. CL with higher light intensity could promote lettuce biomass accumulation, which was attributed to a higher specific leaf FW rather than to leaf area. The oxidative stress degree reflected by ROS production and lipid peroxidation was progressively enhanced by increasing the light intensity and duration of CL. Meanwhile, antioxidant capacity was promoted by higher light intensity, as reflected in greater AsA and GSH pool sizes, stronger AsA synthesis capacity (GalLDH activity) and higher activities of antioxidant enzymes, including APX, DHAR, MDHAR and GR. Among them, APX presented the maximum response to light intensity and the strongest correlation with oxidative stress indexes (H_2_O_2_, O_2_·^−^, and MDA). Chlorophyll fluorescence parameters suggested that reversible photoinhibition induced by higher light intensity emerged at the early phase of CL, but not at the later stage of CL (12 days), with an adaptive increase in light utilization efficiency. However, the aggravation of oxidative stress under higher light intensity could not be prevented with increasing CL duration. In general, CL at a low light intensity (100 µmol·m^−2^·s^−1^) did not induce photooxidative damage in lettuce, as shown by the low amounts of ROS and MDA along with the normal *F*_v_/*F*_m_ value, indicating that photooxidative stress induced by CL can be attributed to excess DLI instead of circadian asynchrony.

## Data Availability Statement

All datasets [generated/analyzed] for this study are included in the manuscript files.

## Author Contributions

WL was the recipient of funds. WL and LZ conceived the experiment. LZ, YZ, CZ, and MS prepared the plant materials, collected samples, and undertook experiments. LZ analyzed the data and prepared the manuscript. WL and LZ revised the manuscript.

## Funding

This research was financed by the National Natural Science Foundation of China (NSFC) (No. 31672202) and Central Public-interest Scientific Institution Basal Research Fund (No. BSRF201711).

## Conflict of Interest

The authors declare that the research was conducted in the absence of any commercial or financial relationships that could be construed as a potential conflict of interest.
